# Milk microbiome and bacterial load following dry cow therapy without antibiotics in dairy cows with healthy mammary gland

**DOI:** 10.1038/s41598-017-08790-5

**Published:** 2017-08-14

**Authors:** Erika C. R. Bonsaglia, Marilia S. Gomes, Igor F. Canisso, Ziyao Zhou, Svetlana F. Lima, Vera L. M. Rall, Georgios Oikonomou, Rodrigo C. Bicalho, Fabio S. Lima

**Affiliations:** 1 Department of Veterinary Clinical Medicine, University of Illinois Urbana-Champaign, Champaign, IL USA; 2 Department of Microbiology and Immunology, Institute of Biosciences, São Paulo State University, Botucatu, Brazil, USA; 3 Department of Population Medicine and Diagnostic Sciences, Cornell University, Ithaca, NY USA; 4 Department of Epidemiology and Population Health, Institute of Infection and Global Health, University of Liverpool, Leahurst, Neston, UK

## Abstract

Preventive infusion of antibiotics in the mammary gland of cows consumes 11 tons/year of medically relevant antimicrobials, yet, this practice might not be critical to prevent new infections in the healthy mammary gland of cows. Here, we used next-generation sequencing and quantitative real-time PCR to determine the impact of dry cow therapy without antibiotics on milk microbiome and bacterial load, respectively. Cows diagnosed as negative for mastitis at dry off were randomly allocated to receive antibiotic (intramammary ceftiofur hydrochloride) and teat sealant or just teat sealant. Firmicutes was the most abundant phylum, and *Corynebacterium, Acinetobacter*, and *Staphylococcus*, often involved in mastitis cases, were the most abundant genera across treatments and time. However, there were no effects of antimicrobial on milk microbiome and bacterial load. Bacterial load was greater at seven days postpartum than at dry off. Dry cow therapy based on teat sealant without antibiotics can be used with no detrimental impacts on milk microbiome and bacterial load in cows with a healthy mammary gland.

## Introduction

Mastitis is the most prevalent disease and the primary cause of economic losses in dairy cows^1^. The major losses caused by mastitis include reduced milk production, discarded milk, premature culling, reduced conception rates, and cost with therapy^2–5^.

New bacterial infections occur more often during the dry-off period than in any other time points during the lactation^6^. Studies have shown that between 13% and 35% of quarters are subclinically infected during dry off period and between 8% and 25% develop intramammary infections in that period^7–9^. Currently, blanket dry cow therapy is the strategy used to control udder health during the dry period in more than 90% of dairy operation in the United States^10^. Blanket dry cow therapy consists of treating dairy cows at dry-off with long-acting intramammary antibiotics. This therapeutic approach has been recommended by the National Mastitis Council as an integral part of its mastitis prevention program^11^.

However, the widely-spread use of blanket dry cow therapy translates into 11 tons of medically important antimicrobials being used intramammary annually^12, 13^. The indiscriminate use of antibiotics in farm animals is often associated with the development of antimicrobial resistance (AMR) and dissemination^14^. The non-judicious use of antibiotics can cause loss of effectiveness that in turn undermine the ability to fight infectious diseases threatening human and animal health.

Although the reduction in antimicrobial use in animals may not directly prevent evolving AMR, it may delay further development and spread, without adversely affecting animal production^15^. Recent studies have indicated that selective dry cow therapy based on the use of on-farm culture and teat sealant alone may allow reduction of antimicrobials without increasing new intramammary infections and somatic cell counts in the following lactation^16–18^. However, the impact of this practice on milk microbiome, bacterial load, and pathogens associated with mastitis remains to be determined.

Development of AMR following treatment with antibiotics is not exclusive of pathogenic bacteria, but it may occur in the ‘innocent’ bystanders (i. e., microbiome makeup) of body systems including the mammary gland^19, 20^. Indeed, mastitis and potentially the dissemination of AMR might be a result of a dysbiosis in the mammary gland^21–23^. In humans, the normal commensal mammary gland microflora protects against *Staphylococcus aureus* ^24^. Therefore, it is reasonable to suggest that commensals may play an important role in mastitis pathogenesis and development of AMR in dairy cows. A recent study evaluated the effects of mastitis treatment with a third-generation cephalosporin (ceftiofur hydrochloride) in the milk microbiome of dairy cows diagnosed with mastitis. The findings of the study revealed that milk microbiome, cure rates, and bacterial load did not shift with the treatment of mastitis suggesting that microbial infections in the mammary gland might not necessarily benefit from antimicrobial use^25^.

Investigation of dry cow therapy without antibiotics effects on mammary gland pathogens and its co-inhabitant microbes are necessary to elucidate mastitis pathogenesis, AMR, and the feasibility of implementing programs to reduce antimicrobial use in dairy cows. Metagenomic approaches are allowing in-depth comparative analyses of multiple sites within an individual and across populations at an affordable price^26^. Studies have been carried out to characterize the milk microbiome of dairy cows with healthy mammary gland and mastitis^21–23, 25, 27^. However, the effects of using dry cow therapy with teat sealant alone or with antibiotics on milk microbiome and bacterial load have not been investigated.

Therefore, the aims of this study were to compare the effects of dry cow therapy with and without antimicrobial, on milk microbiome and bacterial load in cows diagnosed as negative for mastitis at dry off.

## Results

### Descriptive Evaluation of Mastitis Incidence, Somatic Cell Count, and Bacterial Culture

At the time of enrollment (Dry off), there were no differences (*P* = 0.47) in somatic cell count scores between cows assigned to receive antibiotic (ceftiofur hydrochloride) with teat sealant (ATS, score 2.6, n = 36 cows) and cows receiving the teat sealant alone (TS, score 2.5, n = 36 cows). Inclusion of ceftiofur hydrochloride as part of the dry cow therapy did not affect the incidence of mastitis in the first 60 days postpartum (ATS = 8.0% vs. TS = 12.0%, *P* = 0.46), somatic cell count score (ATS = 2.5 vs TS = 2.7, *P* = 0.40), and percent of culture positive cows at day 7 postpartum (ATS = 20% vs TS = 25%, *P* = 0.68).

### Sequencing Results

Milk samples were pooled from all quarters at dry off and seven days postpartum. Within each group, 21 (out of 36) were randomly selected to be sequenced. This design resulted in 84 samples collected individually to assess the microbiome by amplification and next-generation sequencing of the V4 region of the 16s rRNA gene. A single run was performed using the MiSeq sequencer (Illumina, Inc., San Diego, CA) and the V2 chemistry kits (300-cycles) and 84 barcoded samples. Sequences were filtered for size, quality, and for the presence of chimeras.

### Number of reads, richness, diversity, and 16S rRNA gene copy numbers

**Figure 1:**
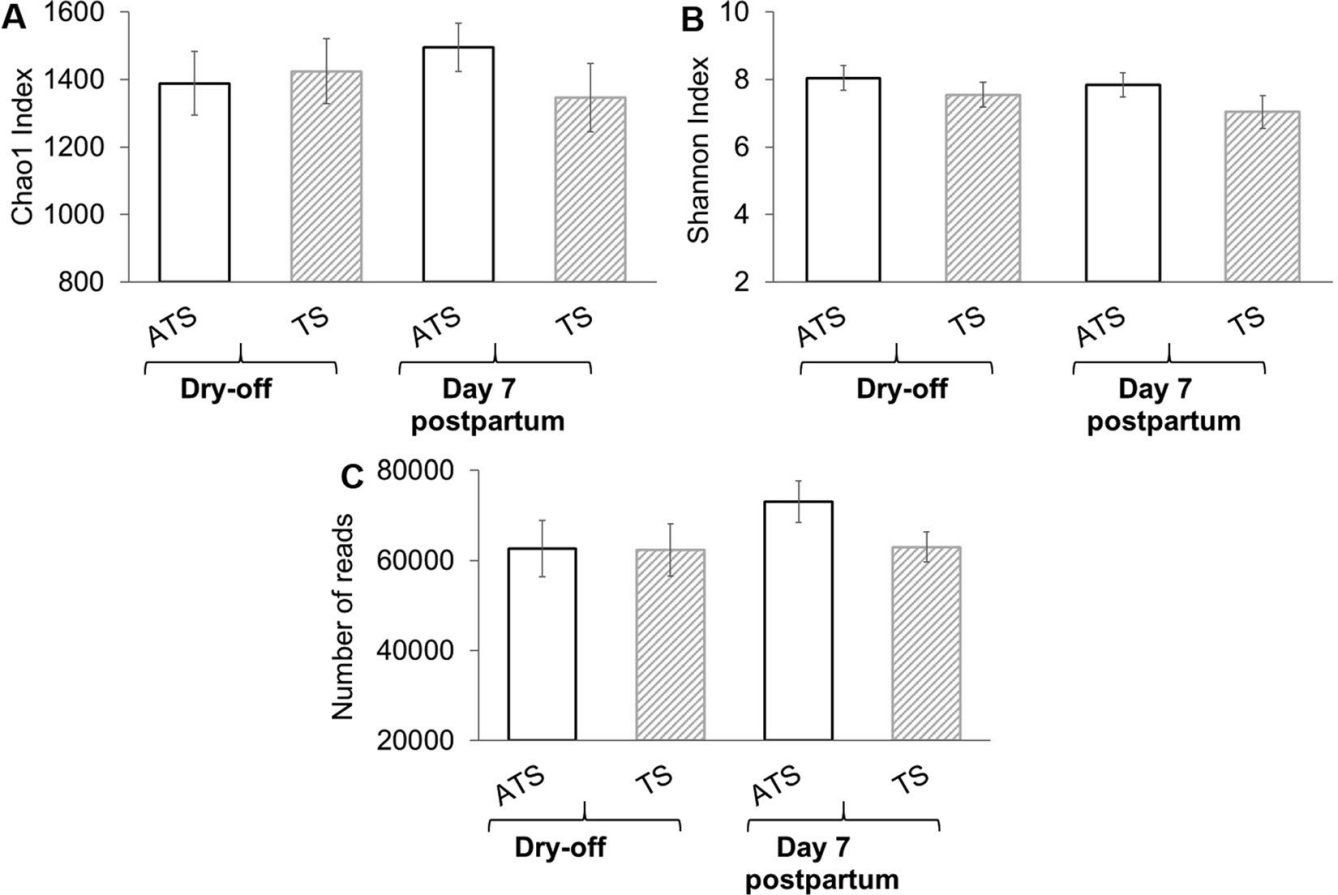
Bar graphs illustrating the Chao1 richness index (**A**), Shannon diversity index (**B**) and number of reads (**C**) for dry cow therapy with antibiotic (ceftiofur hydrochloride) and teat sealant (ATS) or just teat sealant (TS) at dry off and 7 d postpartum. Error bars correspond to standard error of the mean.

The mean number of reads for each group (ATS and TS) was not significantly different at dry off and 7 days postpartum as shown in Fig. 1A. Likewise, no significant differences were observed for OTU richness and alpha diversity indexes, represented by the mean Chao1 richness index illustrated in Fig. 1B. The mean Shannon richness and diversity indexes for each treatment at dry off and 7 days postpartum were also not different as shown in Fig. 1C. Chao1 richness index is a nonparametric estimator of minimum richness. This index is based on the number of rare OTUs within a single sample^28^. When a sample exhibits many singletons, it is likely that more undetected OTUs exist, and the Chao 1 richness index estimates a higher richness than it would estimate for a sample without rare OTUs. Whereas, Shannon diversity index includes both richness and abundance in a single value of evenness. As consequence of these definitions, microbiomes numerically dominated by one or few organisms have low evenness, and when abundance is distributed equally among organisms, the microbiome has high evenness. The richness and the evenness were analyzed to assess whether any divergence was observed across groups and time. The Chao 1 and Shannon indexes were not statistically different between ATS and TS groups, regardless of time points (Fig. 1B and C).

A negative correlation was detected between the total bacterial load, as assessed by the number of 16S rRNA gene copies, and the Shannon diversity index (r = − 0.29, *P*-value < 0.001). Similar negative correlations were found when data were stratified by the occurrence of clinical mastitis, subclinical mastitis, and culture on day 7 postpartum (Table 1). No correlation was found between the number of 16S rRNA gene copies and the Chao 1 richness index (data not shown).

**Table 1:** Correlation between Shannon diversity index and bacterial load in mammary gland health status.

Health status	Correlation	*P*-values
Healthy	−0.33	<0.001
Clinical mastitis	−0.62	0.13
Subclinical mastitis at day 7 postpartum	−0.36	0.005
Culture positive at day 7 postpartum	−0.50	0.07

The bacterial load was measured via proxy of the number of 16S rRNA genes.

Quantitative real-time PCR was used to monitor the amplification of the 16S rRNA-targeted gene during PCR. As a result of this method, an absolute quantification that gives the exact number of the target DNA molecules within a sample, by comparison with DNA standards (serial dilution of our 16S rRNA gene clone) using a calibration curve is provided.

**Figure 2:**
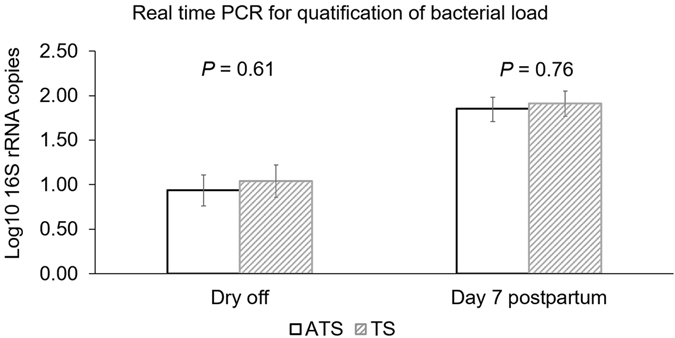
Bar graph illustrating the bacterial load measured as mean log10 number of the 16S rRNA gene identified in milk samples of cows enrolled either on with antibiotic (ceftiofur hydrochloride) and teat sealant (ATS) or just teat sealant (TS) at dry off and 7 days postpartum. Error bars correspond to standard error of the mean.

**Figure 3:**
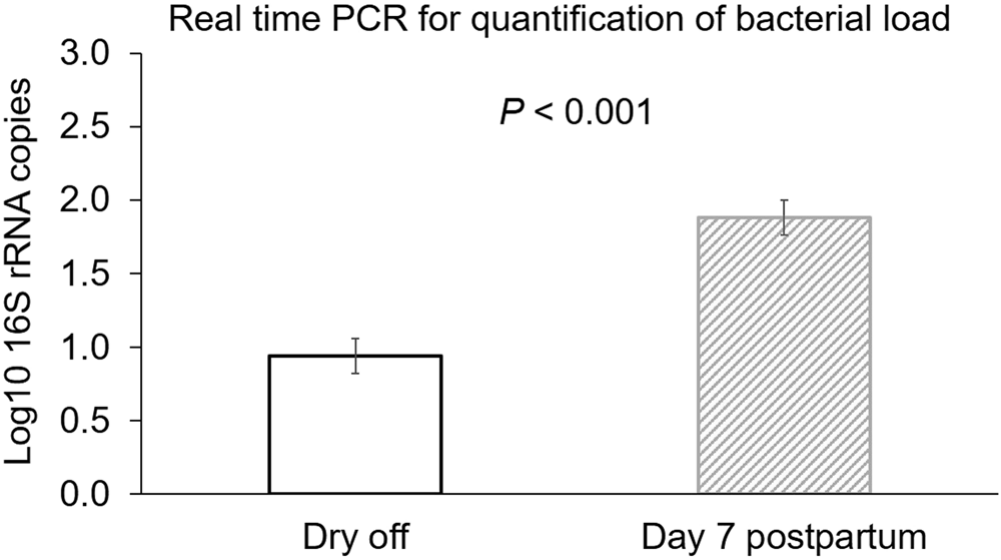
Bar graph illustrating the mean log10 number of the 16S rRNA gene identified in milk samples collected at dry off and 7 days postpartum. Error bars correspond to standard error of the mean.

No significant differences were found between the bacterial load for conventional dry cow therapy (*P* = 0.61) and selective dry cow therapy (*P* = 0.76) at dry off and seven days postpartum (Fig. 2). However, bacterial load in milk increased from the dry off to seven days postpartum (Fig. 3).

### Microbial phylum analysis

**Figure 4:**
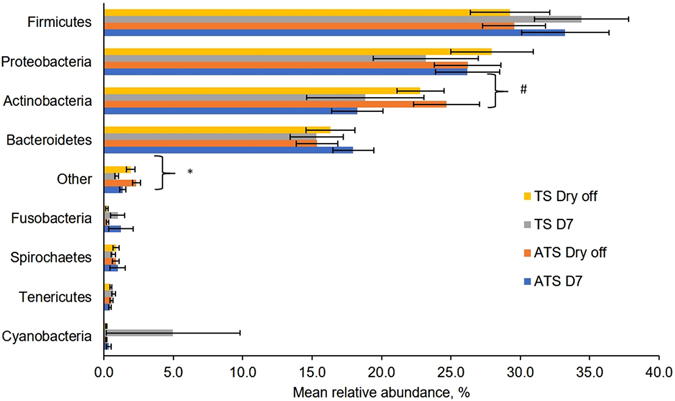
Bar graph illustrating the mean relative abundance of major phylum types either on cows receiving dry cow therapy with antibiotic (ceftiofur hydrochloride) and teat sealant (ATS) or dry cow therapy only with teat sealant (TS) at Dry off and 7 days postpartum. Error bars correspond to standard error of the mean. *P < 0.05 for time effect. ^#^ *P* < 0.10 and *P* > 0.05 for time effect.

The relative abundances of the eight most common phyla of the milk samples regardless of time and treatment include Firmicutes, Proteobacteria, Actinobacteria, Bacteroidetes, Cyanobacteria, Tenericutes, and Fusobacteria. As depicted in Fig. 4. Firmicutes and Proteobacteria were consistently the most abundant phylum across time points and treatments (Fig. 4). The mean relative abundance of the phylum Actinobacteria tended to be reduced (*P* = *0.06*) in the milk samples at 7 days postpartum when compared with samples harvested at the dry off (Fig. 4). However, there were no differences (*P* = 0.81) between treatments for the mean relative abundance of Actinobacteria. The compiled number of phyla in the milk microbiome with minor prevalence named “other” was reduced at day 7 postpartum when compared with the dry period (Fig. 4). No other differences were observed for treatment and time for other phyla.

### Bacterial genera analysis

The prevalence (% of animals detected with the corresponding genus) of the 30 most abundant bacterial genera present in the milk samples regardless of time and treatment is depicted in Table 2. The mean relative abundance for the 30 most common bacterial genera found throughout the different time points according to each treatment is presented in Table 1. *Corynebacterium, Acinetobacter, Arthrobacter, Staphylococcus, and Psychrobacter* were the top 5 most prevalent genera across treatments (Table 2).

**Table 2:** Descriptive statistics of the 30 most abundant bacterial genera.

Bacterial genera	ATS dry off		ATS 7 d pp		TS dry off		TS 7 d pp	
	MRA	P	MRA	P	MRA	P	MRA	P
*Corynebacterium*	13.6	100.0%	9.6	100.0%	11.8	100.0%	13.4	100.0%
*Acinetobacter*	11.1	100.0%	13.2	100.0%	11.3	100.0%	11.1	100.0%
*Arthrobacter*	5.1	100.0%	6.3	100.0%	4.9	100.0%	5.2	100.0%
*Staphylococcus*	4.5	100.0%	1.9	100.0%	4.0	100.0%	5.2	100.0%
*Psychrobacter*	13.6	100.0%	9.6	100.0%	10.9	100.0%	13.4	100.0%
*5–7N15*	3.5	100.0%	2.9	100.0%	2.4	95.2%	3.8	100.0%
*Chryseobacterium*	1.8	100.0%	2.7	100.0%	2.5	100.0%	2.2	100.0%
*Coxiella*	1.1	100.0%	2.3	100.0%	2.0	100.0%	2.8	100.0%
*Facklamia*	3.5	100.0%	1.4	95.2%	1.6	100.0%	1.4	95.2%
*Paracoccus*	2.7	100.0%	2.1	100.0%	0.6	100.0%	1.1	95.2%
*Prevotella*	1.4	100.0%	1.4	100.0%	1.8	100.0%	1.3	100.0%
*Pseudomonas*	1.6	90.5%	1.9	100.0%	1.2	100.0%	1.9	100.0%
*Treponema*	0.0	38.1%	0.0	42.9%	3.3	61.9%	0.1	52.4%
*Paenibacillus*	1.4	95.2%	1.6	100.0%	0.9	100.0%	2.3	9.2%
*Ruminobacter*	0.9	100.0%	1.9	100.0%	1.3	90.5%	0.8	90.5%
*Wautersiella*	0.3	80.9%	0.3	80.9%	2.9	80.9%	0.2	71.4%
*Cellvibrio*	0.8	95.2%	1.2	100.0%	1.3	95.2%	1.0	100.0%
*Sphingobacterium*	1.2	90.5%	1.4	100.0%	0.8	95.2%	1.6	100.0%
*CF231*	1.0	95.2%	1.6	100.0%	0.8	100.0%	1.2	100.0%
*Ruminococcus*	0.8	100.0%	1.1	100.0%	1.1	100.0%	1.3	100.0%
*Brachybacterium*	1.1	90.5%	1.1	100.0%	0.9	90.5%	1.1	90.5%
*Aerococcus*	1.0	100.0%	1.3	100.0%	1.0	100.0%	1.4	100.0%
*Coprococcus*	1.8	95.2%	0.4	95.2%	1.7	95.2%	0.5	90.5%
*Luteimonas*	0.4	71.4%	1.5	85.7%	0.6	85.7%	1.1	95.2%
*Fusobacterium*	0.4	80.9%	1.3	90.5%	0.2	85.7%	1.6	76.2%
*Porphyromonas*	0.9	100.0%	1.3	100.0%	0.8	100.0%	1.2	100.0%
*Clostridium*	1.1	100.0%	1.0	95.2%	1.1	90.5%	0.7	95.2%
*Dorea*	0.3	85.7%	1.5	100.0%	0.3	95.2%	1.2	95.2%
*Bacteroides*	1.0	100.0%	0.9	100.0%	1.0	100.0%	0.8	100.0%
*Bacillus*	1.2	100.0%	0.9	95.2%	0.9	95.2%	0.6	85.7%

^1^MRA = Mean relative abundance of bacterial genera at dry off and 7 days postpartum for cows receiving antibiotic (ceftiofur hydrochloride) and teat sealant (ATS) or just teat sealant (TS). ^2^P = Percentage of study milk samples in which the indicated genus was detected at the given sample. pp = postpartum.

**Figure 5:**
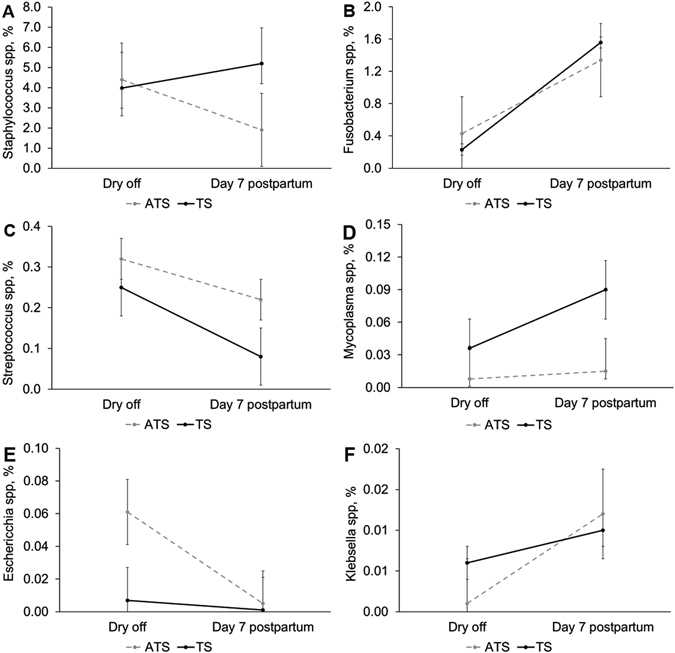
Mean relative abundance of the genus Staphylococcus (**A**), Fusobacterium (**B**), Streptococcus (**C**), Mycoplasma (**D**), Escherichia (**E**), and Klebsiella (**F**) according to time of sample collection (dry off and day 7 postpartum) in cows receiving dry cow therapy with antibiotic (ceftiofur hydrochloride) and teat sealant (ATS) or only teat sealant (TS). Error bars correspond to standard error of the mean.

We performed a multivariable analysis to compare the mean relative abundance for bacterial genera known as major mastitis pathogens (*Escherichia spp., Klebsiella spp., Mycoplasma spp., Staphylococcus spp., and Streptococcus spp*.). The use teat sealant with or without ceftiofur hydrochloride at dry off did not affect the mean relative abundance of these major genera of mastitis-associated pathogens (Fig. 5A–F).

### Multivariate Analysis of Microbiome Data

**Figure 6:**
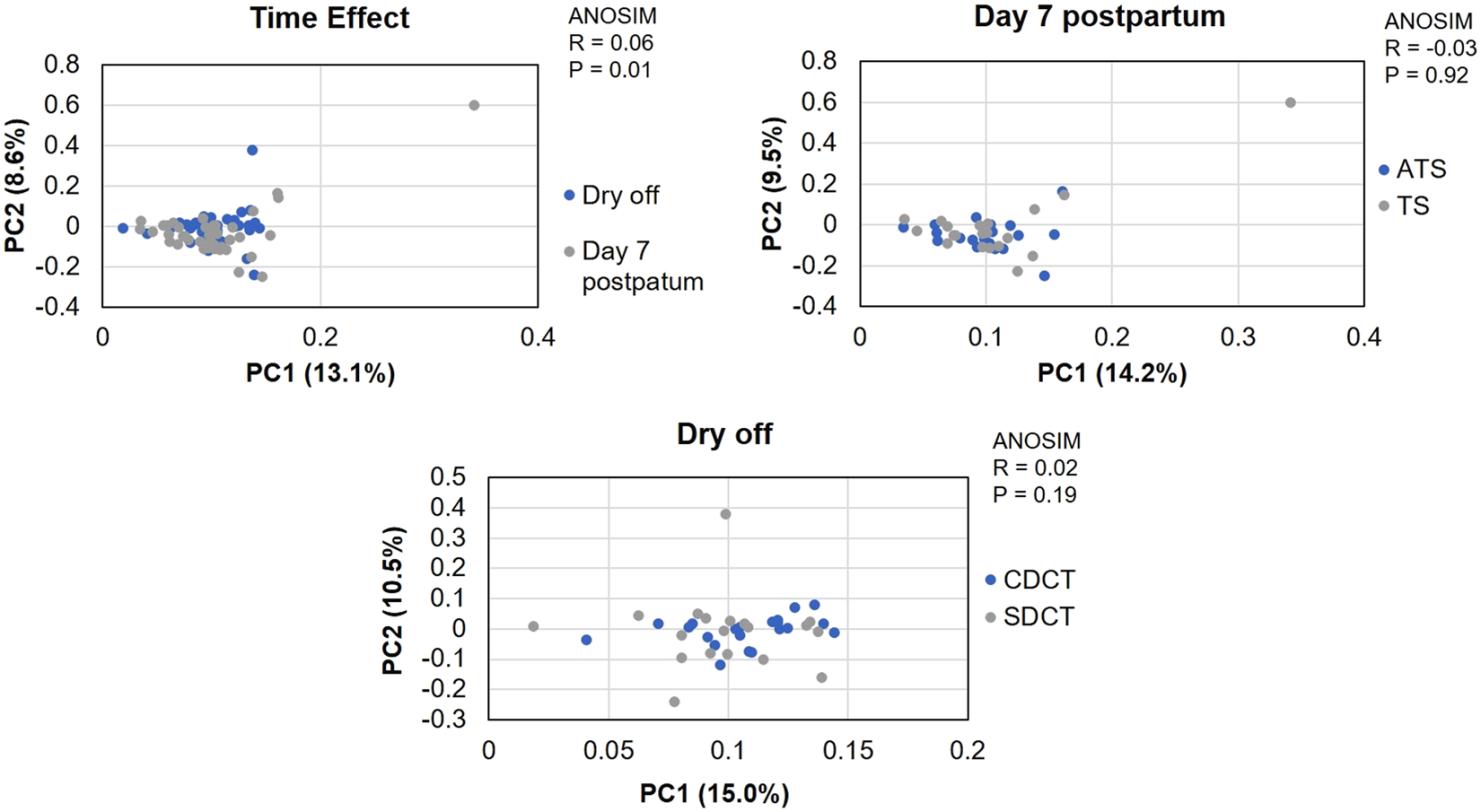
Principal component analysis of weighted Unifrac distances and ANOSIM analysis comparing the microbiome data per time (**A**) of sample collection (dry off and day 7 postpartum) and for conventional dry cow therapy with antibiotic (ceftiofur hydrochloride) and teat sealant (ATS) or selective dry cow therapy receiving only teat sealant (TS) at Dry off (**B**) and 7 days postpartum (**C**).

Analysis of similarities (ANOSIM) and principal component analysis (PCA) revealed that no major differences of treatment at dry off (Fig. 6A) or day 7 postpartum (Fig. 6B) were identified in current study. Nonetheless, the ANOSIM revealed that microbiome based on the 50 most prevalent genera was different between dry off and day 7 postpartum as depicted in Fig. 6C.

## Discussion

The purpose of the current study was to assess the effects of dry cow therapy, with antibiotics and teat sealant or teat sealant alone on milk microbiome and bacterial load. Our findings suggest that cows screened negative for mastitis at the dry off can be managed with teat sealant alone without detrimental effects on milk microbiome and bacterial load at first week postpartum. Rapidly-evolving AMR is a global concern to human health and the food supply chain^29, 30^. Indeed, AMR has increased healthcare costs in US$ 4–5 billion per year in the United States^31^. Prophylactic and metaphylactic use of antibiotics in livestock are a major concern for AMR^32, 33^. Thus, our results suggest an alternative for a rational use of antimicrobial in livestock.

Blanket dry cow therapy has been used to reduce new intramammary infection and to treat cases of chronic mastitis caused by *Staphylococcus aureus* and *Streptococcus agalactiae*. On the other hand, blanket dry cow therapy exposed cows without a history of mammary gland infection to unnecessary antimicrobial use. Recent studies out of Europe and Canada demonstrated benefits of selective dry cow therapy without major detrimental effects on the occurrence of new intramammary cases and somatic cells count^16–18^. The current study was performed to understand the impact of ceftiofur hydrochloride at dry off on mammary gland dysbiosis, presence of pathogens on the makeup of milk microbiome, and bacterial load.

Dysbiosis, defined as breakdown in the balance between putative microbial commensals and pathogens, has been suggested to contribute to mastitis pathogenesis and the dissemination of AMR through milk^21–23^. Blanket dry cow therapy can disturb the microbiome of a healthy mammary gland. The current study explored the possibility that eliminating ceftiofur hydrochloride at the dry off in cows without mastitis would prevent dysbiosis, favoring the maintenance of commensals that help protect the mammary gland from pathogens like observed in humans^24^. However, inclusion of ceftiofur hydrochloride did not shift milk microbiome. The absence of shift in milk microbiome may indicate that microbial communities are dynamic, and an antimicrobial induced disturbance of the milk microflora could be reversed by the time that cows started a new lactation (i.e., ~60d later). This potential explanation is consistent with a recent study revealing that milk microbiome returned to previous composition 14d after experimentally induced mastitis with *E. coli* and treatment with ceftiofur^25^. A second possibility is that ceftiofur hydrochloride disturbances in the healthy mammary gland are negligible not leading to the occurrence of dysbiosis. In the present study, only healthy cows were used, thus, the results in milk microbiome shift could have been different if cows with mastitis were also included. The apparent lack of microbiome shift could be due to the variability of individual cow’s milk microbiome, the low abundance of minor taxa, and the lack of specificity of analysis based on phylum and genus.

Blanket dry cow therapy presumably prevents and treats mastitis caused by *Staphylococcus aureus* and *Streptococcus agalactiae* at dry off. These major mastitis pathogens were not present in the cows enrolled in this study. However, it is vital to emphasize that the cows enrolled in the current study represent the majority of cows in the US^34^, making the use of antimicrobial at dry off a questionable practice. The current dataset corroborates Cameron *et al*.^16^ findings that revealed similar prevalence of new intramammary infections for cows receiving blanket or selective dry cow therapy based on bacterial culture performed at the farm. Thus, intramammary antibiotic infusion at the dry off does not seem to be a necessary to protect the mammary gland of healthy cows.

A second major contributor to the current results was the use of internal teat sealant. This common constituent of dry cow therapy is formulated with bismuth subnitrate into an inert viscous malleable paste without antimicrobial properties. The teat sealant creates a physical barrier that during the dry period helps to prevent entrance of pathogens into the mammary gland. Studies demonstrated that the number of new intramammary infections detected in cows treated with teat sealant alone as dry cow therapy were not different from those cows receiving antimicrobials and teat sealant^16, 17, 35^. We speculate that the physical barrier derived from the use of the internal teat sealant support the maintenance of an environment favorable for commensals in the mammary gland, which in turn may help microbiome stability during the dry period.

*Corynebacterium*, *Acinetobacter*, and *Staphylococcus* were among the most prevalent genus identified in our data analysis. Species from these genera might be involved with the development of mastitis in dairy cows when environmental factors and host immunity are favorable^36–38^. Since 16S rRNA analysis employed herein is based on genus taxonomy, limited conclusions can be drawn for the role of species included in these genera following the treatment with teat sealant alone or with ceftiofur hydrochloride. Some species included in these genera such as *Staphylococcus aureus* have low probability of cure^39, 40^, while others *Staphylococcus sp*. are considered pathogens of minor importance^41, 42^. Additionally, *Staphylococcus* and *Corynebacterium* interact synergistically in the human milk against the benign microbiota to develop mastitis^43^. Likewise, mastitis-associated pathogens might disturb milk microbiome in a manner that the current experimental design did not allow us to investigate, although this was outside of the scope of this study.

Firmicutes was the most abundant phylum in our study, followed by Proteobacteria, Actinobacteria, and Bacteroidetes. Firmicutes denotes an important bacterial group for the milk microbiota^22, 44^, but its specific role of milk microbiome remains to be determined. Fusobacterium phylum was more prevalent at 7 d postpartum than at dry off. This bacterium is present in the environment and in milk samples of cows with mastitis, but it does not appear to be directly responsible for the mastitis^21^. Concurrent occurrence of Fusobacterium with mastitis-associated pathogens in early lactation might be due to ongoing changes during the initiation of lactation^21^.

Shannon diversity and Chao1 richness indexes were not significantly different between cows receiving antibiotic and teat sealant or teat sealant alone. These results contrast with a previous study identifying variation in alpha diversity within 14 days following antimicrobial treatment of cows with experimentally induced mastitis^25^. The discrepancy between the referred^25^ and our study could be attributed to the inclusion of mastitic cows and duration of antimicrobial treatment (>60d vs. 14d). Moreover, a negative correlation was observed between the Shannon diversity index and bacterial load indicating that when microbial colonization increases there is a corresponding reduction in microbial diversity. This finding is consistent with a human study having a negative correlation between bacterial load and alpha-diversity index in milk samples^45^. The results of our principal component analysis and analysis of similarities corroborate to the genera, phylum, and pathogens results indicating that there were no effects of using antibiotics during dry off on milk microbiome. The only difference found in our beta diversity were the effects of time in our analysis of similarities suggesting that stage of lactation might a more important factor influencing milk microbiome composition than the use of antibiotics at dry off.

## Conclusion

Use of dry cow therapy based on a standalone teat sealant without mastitis resulted in a similar incidence of mastitis, somatic cell count, bacterial culture, bacterial load and milk microbiome than cows assigned to receive teat sealant with antibiotic. The current findings suggest that omitting antibiotics from dry cow therapy has no detrimental effects in cows without mastitis at the dry off. Future studies need to investigate the impact of dry cow therapy strategies (with or without antibiotics or teat sealant) on milk resistome and AMR dissemination.

## Methods

### Ethics statement

The experimental protocol (#15060) was approved by the Institutional Animal Care and Use Committee at the University of Illinois at Urbana-Champaign. The methods were carried out in accordance with the approved guidelines.

### Experimental design, housing, sampling, and enrollment criteria

The study was conducted in a single dairy farm located at the University of Illinois-Urbana. Cows were housed in sand-bedded free stall barn during the prepartum period and tie-stall barn during the first 60 days of the postpartum period. The selection criterion for pre-enrollment involved the collection of milk samples 24 hours’ prior the day assigned for dry off and evaluation of bacterial growth using a commercial on farm-culture system (Accumast®, FERA Animal Health, Ithaca, NY). Only cows that had no clinical signs for mastitis or bacterial growth were eligible to be enrolled in the study. Multiparous Holstein cows (n = 72) were randomly allocated to either receive an intramammary infusion of 500 mg of ceftiofur hydrochloride (Spectramast DC, Zoetis Inc. Kalamazoo, MI, USA) and a teat sealant (Orbeseal®, Zoetis Inc. Kalamazoo, MI, USA) in each of the 4 quarters (ATS, n = 36); or to remain as untreated control only receiving the teat sealant (TS, n = 36).

Previously to sampling, cows were properly prepared for milking and milked in the afternoon of the dry off day. Thereafter, the teats were cleaned with a gauzed soaked in 70% alcohol, 1 to 5 ml of milk samples aseptically collected from each quarter. Within cow, samples from each quarter were combined into in a 50-mL graded sterile conical tube (Corning Life Sciences, Tewksbury, MA) for further analyses. Milk samples were placed directly into a cooler with ice and brought to the laboratory for bacterial culture. Then, 0.1 ml was plated in on-farm culture petri dish with specific media (Accumast^®^, FERA Animal Health, Ithaca, NY) using sterile cotton-tipped swabs. Inoculated plates were incubated at 37 °C for 16 h and then evaluated for the presence of common mammary gland pathogens as follows: *Escherichia coli*, *Klebsiella* spp., *Pseudomonas* spp., *Streptococcus* spp., *Streptococcus uberis*, *Enterococcus* spp., *Lactococcus lactis*, *Staphylococcus* spp., *Staphylococcus haemolytic*, and *Staphylococcus aureus*. As aforementioned, only cows with negative bacterial culture and free from clinical signs of mastitis were used in the current study. Cows enrolled in the study had milk samples collected at the dry-off and day seven post calving for microbiological evaluation and somatic cell count (SCC). The plates having more than three colonies after 16 h incubation at 37 °C were considered positive and the respective samples were discarded. The assessment of SCC was performed using the DeLaval Cell Counter DCC^®^ (DeLaval Inc. Kansas City, MO). This automated system uses individual disposable cartridges to generate the numbers somatic cell in seconds. The SCC data is presented as somatic count score that was calculated as log_10_ of the amount of cell detected by DeLaval Cell Counter. Additionally, a 2 mL milk aliquot of was stored at −80 °C until further DNA extraction for determination of the cow’s milk microbiome.

### DNA extraction, DNA amplification, and sequencing of bacterial 16S rRNA gene

The DNA was extracted from all samples using PowerSoil DNA Isolation Kit (MO BIO Laboratory Inc., Carlsbad, CA) following the manufacturer’s protocol. The V4 hypervariable region of the bacterial/archaeal 16S rRNA gene was amplified by PCR according to a previously described protocol and optimized for the Illumina MiSeq platform (Illumina Inc., San Diego, CA, USA)^46^ using different 12-bp error-correcting Golay barcodes for the 16S rRNA gene PCR^47^.

The reactions were performed using 10 µM of each primer (515 F and 806 R), EconoTaq Plus Green 1x Master Mix (Lucigen®, Middleton, WI, USA), 5 ng–50 ng of individual metagenomic DNA samples and ultrapure water to bring the final reaction volume to 25 µL. Blank controls in which no DNA was added to the reaction were performed. All reactions were set up in triplicate. The PCR conditions for amplification were: 1) initial denaturing step, 94 °C for 3 min; 2) 35 cycles of 94 °C for 45s; 3) 50 °C for 1 min; 4) 72 °C for 90s; and 5) final elongation step of 72 °C for 10 min. Replicates were pooled, and visualized by electrophoresis using 1.2% (wt/vol) agarose gels stained with 0.5 mg/ml ethidium bromide. The DNA was purified using Gel/PCR Fragments Extraction Kit (IBI Scientific, Peosta, IA, USA). The quantification of purified DNA was carried out using NanoDrop ND-1000 spectrophotometer (NanoDrop Technologies, Rockland, DE, USA). All samples were standardized to the same concentration and pooled for sequencing on the Illumina MiSeq platform (Illumina Inc., San Diego, CA, USA), into two different runs according to specific barcode primers. Final equimolar libraries were prepared and sequenced using the MiSeq Reagent Kit V2–300 cycles.

### Bioinformatics

The 16S rRNA sequences obtained were processed through the open-source pipeline Quantitative Insights into Microbial Ecology (QIIME) version 1.7.0-dev^48^. Sequences were filtered for quality using established guidelines^49^ and were binned into operational taxonomic units (OTUs) based on 97% identity using UCLUST^50^ against the Greengenes reference database^51^ (May 2013 release). Chimeric sequences were removed, and low-abundance clusters were filtered using USEARCH^52^. The representative sequences for each OTU were compared against the Greengenes database for taxonomy assignment, and only full-length, high-quality reads (−r = 0) were used for analysis. Shannon diversity index output was generated by the QIIME pipeline. Before estimating the Shannon index, all sample libraries were rarefied to an equal depth of 10,000 sequences using QIIME.

### Quantitative PCR

Within each group (ATS and TS), 21 (out of 36) cows were randomly selected to have milk samples assessed for bacterial load at dry off and seven days postpartum. For determination of total bacterial load by quantitative PCR (qPCR), a plasmid containing the amplified V6 hypervariable region was cloned into TOP10 cells using a Zero Blunt^®^ TOPO^®^ PCR cloning kit (Life Technologies, Darmstadt, Germany). Plasmid was purified with the QIAprep Spin Miniprep Kit (Qiagen, Valencia, CA, USA) and quantified using Quant-iT™ PicoGreen^®^ and a dsDNA Broad Range Assay Kit (Life Technologies Corporation, Carlsbad, CA). Insertion of the DNA fragment was confirmed by agarose gel electrophoresis, and by sequencing at the Cornell University Life Science Core Laboratories Center. The 16S rRNA copy numbers were measured by qPCR using forward 5′ TGG AGC ATG TGG TTT AAT TCG A 3′, and reverse 5′ TGC GGG ACT TAA CCC AAC A 3′ primers previously described^51^. PCRs were performed in 15 μL volumes composed of 1X iQTMSybr Green Supermix (BIO-RAD Laboratories, Hercules, CA), 300 nM of each primer and 5 pg-50 ng of genomic DNA (or plasmid DNA standards). The thermal cycler conditions were as follows: denaturation at 95 °C for 3 min, 40 amplification cycles (95 °C for 10s, 55 °C for 30s) and two final steps at 95 °C for 1 min and 55 °C for 1 min followed by melting curve determination. All reactions were performed in duplicate (plasmid standards, BTM samples, and blank control) using MyiQTM Real-Time PCR Detection System (BIO-RAD Laboratories, Hercules, CA, USA). The quantification of 16S target DNA was achieved by ten-fold serial dilutions ranging from 100 to 107 plasmid copies of the previously quantified plasmid standard. The average of the cycle threshold value was used for calculation of the bacterial load.

### Statistical analysis

Categorical data were analyzed using the GLIMMIX procedure of SAS (SAS version 9.4; SAS Institute Inc., Cary, NC) fitting a binary distribution. The continuous data with repeated measures over time within an experimental unit were analyzed using the GLIMMIX procedure of SAS (SAS/STAT version 9.4; SAS Institute Inc., Cary, NC) with models fitting a Gaussian distribution. Data were tested for normality of residuals, and non-normally distributed data were transformed before analysis if improvement in residual distribution was observed. The covariance structure that resulted in the smallest Akaike’s information criterion was selected for the model. Cow was used as a random effect in our models.

Shannon and Chao1 indexes output were generated by QIIME pipeline. Before estimating the Shannon and Chao1 indexes, all sample libraries were rarefied to an equal depth of 10,000 sequences using QIIME. Chao1 and Shannon indexes, the total number of reads, and the log of the 16S rDNA copy number (total bacterial load) were analyzed using ANOVA by general linear models fitted in JMP Pro 13 (SAS Institute Inc., Cary, NC).

Dunnett’s multiple comparisons procedure was performed to compare the mean number of reads, Shannon index and Chao 1 for treatments (ATS and TS) at dry off and day seven postpartum. Correlations between total bacterial load and alpha-diversity indexes (Shannon and Chao 1 indexes) were assessed using simple linear regression in JMP Pro 13 software (SAS Institute Inc.). The relative abundances of microbial phyla and genera types in milk samples at dry off and seven days after calving were compared using general linear models fitted in JPM Pro 13 (SAS Institute Inc., Cary, NC).

Dunnett’s multiple comparisons procedure was used to compare the mean relative abundance of the most abundant bacterial phyla and the genera of each treatment at dry off and seven days after calving. Differences with a value of *P* ≤ 0.05 were considered significant and those with a value of 0.05 < *P* ≤ 0.10 were considered tendencies.

Multivariate analysis of microbiome data was carried out using R (R Core Team, Vienna, Austria) and JPM Pro 13 (SAS Institute Inc., Cary NC). Data from the 50 most prevalent genera were used to analyze beta diversity through analysis of similarities (ANOSIM) using non-rarefied data normalized employing the packages vegan in R. Principal component analysis (PCA) based on the 50 most prevalent genera was performed using JPM Pro 13 (SAS Institute Inc).
